# Supplementary material for: An analysis of global legislation and regulation related to drowning prevention
Source: PLOS Glob Public Health. 2026 Mar 25;6(3):e0005337. doi: 10.1371/journal.pgph.0005337 (PMC13016334; doi:10.1371/journal.pgph.0005337)
Supplement: S1 Table — (DOCX) [file pgph.0005337.s001.docx]

**Table S1. Variables and data sources**

| **Data source** | **Variable (renamed)** | **Description** | **Included in final modelling?** |
| --- | --- | --- | --- |
| **WHO global status report** | GDP (PPP per capita) | National gross domestic product (PPP) per capita | Yes |
|  | Drowning mortality rate | Age‐standardized drowning mortality rate per 100 000 population | Yes |
|  | National drowning-prevention strategy | Existence of a national drowning-prevention strategy | Yes |
|  | Disaster-risk policy includes drowning | Whether national disaster-risk policy explicitly addresses drowning | Yes |
|  | Private pool fencing law | Legislation requiring fencing of private swimming pools | Yes |
|  | Public pool fencing law | Legislation requiring fencing of public swimming pools | Yes |
|  | Domestic water-transport safety law | Safety regulations for domestic water transport | Yes |
|  | Lifejacket-use law | Laws mandating life-jacket use in relevant contexts | Yes |
|  | Alcohol restrictions near water | Regulations limiting alcohol consumption near aquatic locations | Yes |
|  | Total legislative measures (global) | Count of “Yes” across six national legislative items (0–6) | Yes |
| **WHO regional reports** | Total legislative measures (regional) | Total regional disaster-risk-reduction legislative provisions (derived) | Yes |
|  | Total enforcement score (regional) | Aggregate enforcement score across all regional items (0–190) (derived) | Yes |
| **WDI climate portal** | Average temperature | Mean surface air temperature (1995–2020) | Yes |
| **V-Dem 2021** | Legislative transparency/enforcement index | V-Dem index of legislative transparency and enforcement | Yes |
|  | Respect for rule of law index | V-Dem index of respect for the rule of law | Yes |
| **World Justice Project** | Rule of Law (WJP) | Overall WJP Rule of Law Index score | Yes |
|  | Regulatory Enforcement (WJP) | WJP Index: Factor 6, regulatory enforcement | Yes |
| **World Bank WDI** | Child population share | % of population aged 0–14 years | No |
|  | Alcohol consumption per capita | Per-capita alcohol consumption (litres pure alcohol) | Yes |
|  | Physicians per 1 000 people | Number of physicians per 1 000 population | No |
|  | Hospital beds per 1 000 people | Number of hospital beds per 1 000 population | No |
|  | Health expenditure per capita | Current health-care expenditure per capita (USD) | No |
|  | Annual precipitation | Annual precipitation (mm) | No |
|  | Total population | Total national population | No |
|  | Tourism intensity | Annual international arrivals per 1 000 population | No |
| **Global Health Security (2021)** | Overall GHS score | Composite GHS Index score | No |
|  | Health-sector capacity | GHS: robustness of health-sector capacity | Yes |
|  | Commitments to capacity/financing | GHS: commitments to capacity, financing, norms adherence | No |
|  | Risk environment | GHS: vulnerability to biological threats | No |
|  | Government effectiveness | GHS subscore for government effectiveness | No |
|  | Infrastructure adequacy | GHS subscore for infrastructure adequacy | No |
|  | Environmental risks | GHS subscore for environmental bio-risk | No |
|  | Urbanisation | GHS subscore for urbanisation | Yes |
|  | Natural-disaster risk (rev.) | GHS subscore for natural-disaster risk (reverse scored) | Yes |
|  | Access to water & sanitation | GHS subscore for access to potable water and sanitation | Yes |
|  | Public-health spending per capita | GHS subscore for per-capita public-health spending | Yes |
