# Supplementary material for: An analysis of global legislation and regulation related to drowning prevention
Source: PLOS Glob Public Health. 2026 Mar 25;6(3):e0005337. doi: 10.1371/journal.pgph.0005337 (PMC13016334; doi:10.1371/journal.pgph.0005337)
Supplement: S2 Table — (DOCX) [file pgph.0005337.s002.docx]

**Table S2. Outcome summaries by model**

|  | **Variable** | **n** | **n miss** | **Mean** | **SD** | **Min** | **Q1** | **Median** | **Q3** | **Max** |
| --- | --- | --- | --- | --- | --- | --- | --- | --- | --- | --- |
| M0 | Deaths | 113 | 14 | 2050.133 | 7784.517 | 0.0 | 66.00 | 218.00 | 788.00 | 56494.0 |
|  | Rate per 100,000 | 115 | 12 | 3.775 | 3.696 | 0.0 | 1.15 | 2.80 | 5.65 | 28.1 |
|  | Drowning rate | 115 | 12 | 3.775 | 3.696 | 0.0 | 1.15 | 2.80 | 5.65 | 28.1 |
| M1 | Deaths | 92 | 12 | 2282.359 | 8583.606 | 1.0 | 54.75 | 167.50 | 796.75 | 56494.0 |
|  | Rate per 100,000 | 94 | 10 | 3.715 | 3.781 | 0.2 | 1.10 | 2.65 | 5.35 | 28.1 |
|  | Drowning rate | 94 | 10 | 3.715 | 3.781 | 0.2 | 1.10 | 2.65 | 5.35 | 28.1 |
| M2 | Deaths | 113 | 14 | 2050.133 | 7784.517 | 0.0 | 66.00 | 218.00 | 788.00 | 56494.0 |
|  | Rate per 100,000 | 115 | 12 | 3.775 | 3.696 | 0.0 | 1.15 | 2.80 | 5.65 | 28.1 |
|  | Drowning rate | 115 | 12 | 3.775 | 3.696 | 0.0 | 1.15 | 2.80 | 5.65 | 28.1 |
