# Supplementary material for: An analysis of global legislation and regulation related to drowning prevention
Source: PLOS Glob Public Health. 2026 Mar 25;6(3):e0005337. doi: 10.1371/journal.pgph.0005337 (PMC13016334; doi:10.1371/journal.pgph.0005337)
Supplement: S3 Table — (DOCX) [file pgph.0005337.s003.docx]

**Table S3. Countries included in main models**

| **Country** | **M0** | **M1** | **M2** | **Drowning Rate** | **Legislation Count** | **V-DEM: Transpar-ency** | **V-DEM: Rule of Law** | **WJP: Reg. Enforce-ment** | **WJP: Rule of Law** |
| --- | --- | --- | --- | --- | --- | --- | --- | --- | --- |
| **Albania** | ✓ | ✓ | ✓ | 0.9 | 3 | 1.02 | 0.58 | 0.43 | 0.48 |
| **Argentina** |  | ✓ | ✓ | 0.7 | 4 | NA | NA | 0.48 | 0.56 |
| **Armenia** | ✓ | ✓ | ✓ | 1.1 | 0 | 1.11 | 0.79 | NA | NA |
| **Australia** | ✓ | ✓ | ✓ | 0.8 | 7 | 2.52 | 3.38 | 0.82 | 0.80 |
| **Austria** | ✓ | ✓ | ✓ | 0.4 | 2 | 1.46 | 2.62 | 0.80 | 0.80 |
| **Bahamas** | ✓ | ✓ | ✓ | 6.4 | 1 | NA | NA | 0.51 | 0.59 |
| **Bahrain** | ✓ | ✓ | ✓ | 1.0 | 5 | -0.61 | -1.08 | NA | NA |
| **Bangladesh** | ✓ | ✓ | ✓ | 5.7 | 1 | -1.22 | -1.88 | 0.38 | 0.38 |
| **Belarus** | ✓ | ✓ | ✓ | 3.8 | 4 | -2.40 | -1.49 | 0.44 | 0.45 |
| **Belgium** | ✓ | ✓ | ✓ | 0.6 | 2 | 2.53 | 2.87 | 0.79 | 0.78 |
| **Belize** | ✓ | ✓ | ✓ | 4.0 | 2 | NA | NA | 0.41 | 0.49 |
| **Benin** | ✓ | ✓ | ✓ | 7.7 | 2 | 0.02 | 1.70 | 0.48 | 0.48 |
| **Bermuda** |  | ✓ | ✓ | NA | 3 | NA | NA | NA | NA |
| **Bhutan** | ✓ | ✓ | ✓ | 3.0 | 0 | 1.79 | 1.67 | NA | NA |
| **Bolivia** | ✓ | ✓ | ✓ | 2.4 | 3 | 1.25 | 0.66 | 0.39 | 0.37 |
| **Brazil** | ✓ | ✓ | ✓ | 2.1 | 5 | 0.39 | 0.47 | 0.48 | 0.49 |
| **Brunei Darussalam** |  | ✓ | ✓ | NA | 0 | NA | NA | NA | NA |
| **Bulgaria** | ✓ | ✓ | ✓ | 1.7 | 4 | 0.48 | 0.07 | 0.53 | 0.56 |
| **Burkina Faso** | ✓ | ✓ | ✓ | 7.4 | 0 | 2.58 | 1.43 | 0.46 | 0.47 |
| **Burundi** | ✓ | ✓ | ✓ | 7.7 | 2 | -1.18 | -0.88 | NA | NA |
| **Cabo Verde** | ✓ | ✓ | ✓ | 7.3 | 4 | 1.05 | 0.86 | NA | NA |
| **Cambodia** | ✓ | ✓ | ✓ | 7.6 | 2 | -1.37 | -1.82 | 0.26 | 0.31 |
| **Canada** | ✓ | ✓ | ✓ | 0.8 | 3 | 2.42 | 1.88 | 0.81 | 0.80 |
| **Chad** | ✓ | ✓ | ✓ | 7.8 | 0 | -0.81 | -3.09 | NA | NA |
| **Chile** | ✓ | ✓ | ✓ | 1.3 | 5 | 2.49 | 1.41 | 0.64 | 0.66 |
| **China** | ✓ | ✓ | ✓ | 4.0 | 5 | -0.95 | -0.62 | 0.49 | 0.47 |
| **Colombia** | ✓ | ✓ | ✓ | 1.3 | 4 | 0.77 | 0.38 | 0.52 | 0.48 |
| **Comoros** | ✓ | ✓ | ✓ | 4.3 | 2 | -0.33 | -0.87 | NA | NA |
| **Congo** | ✓ | ✓ | ✓ | 3.7 | 2 | -0.32 | -0.28 | 0.43 | 0.40 |
| **Cook Islands** |  | ✓ | ✓ | 28.1 | 2 | NA | NA | NA | NA |
| **Costa Rica** | ✓ | ✓ | ✓ | 2.2 | 4 | 2.35 | 2.74 | 0.68 | 0.68 |
| **Croatia** | ✓ | ✓ | ✓ | 1.7 | 3 | 1.10 | 0.63 | 0.56 | 0.61 |
| **Cuba** | ✓ | ✓ | ✓ | 1.4 | 4 | -1.72 | -1.76 | NA | NA |
| **Cyprus** | ✓ | ✓ | ✓ | 1.8 | 3 | 1.74 | 1.49 | 0.66 | 0.68 |
| **Czechia** | ✓ | ✓ | ✓ | 1.4 | 1 | 1.03 | 1.27 | 0.71 | 0.73 |
| **Côte d’Ivoire** | ✓ | ✓ | ✓ | 6.1 | 4 | 0.55 | 0.43 | 0.52 | 0.45 |
| **Denmark** | ✓ | ✓ | ✓ | 0.6 | 3 | 1.86 | 2.75 | 0.88 | 0.90 |
| **Dominica** | ✓ | ✓ | ✓ | 10.5 | 1 | NA | NA | 0.53 | 0.58 |
| **Dominican Republic** | ✓ | ✓ | ✓ | 2.0 | 2 | 0.85 | 0.44 | 0.42 | 0.49 |
| **Ecuador** | ✓ | ✓ | ✓ | 2.7 | 2 | 0.90 | 0.44 | 0.47 | 0.47 |
| **El Salvador** | ✓ | ✓ | ✓ | 8.3 | 4 | -1.99 | -1.16 | 0.48 | 0.45 |
| **Estonia** | ✓ | ✓ | ✓ | 3.8 | 3 | 2.92 | 3.28 | 0.81 | 0.82 |
| **Eswatini** | ✓ | ✓ | ✓ | 9.4 | 0 | -1.28 | -1.04 | NA | NA |
| **Ethiopia** | ✓ | ✓ | ✓ | 3.2 | 0 | 0.03 | -0.58 | 0.36 | 0.38 |
| **Fiji** |  | ✓ | ✓ | NA | 0 | 0.08 | 0.95 | NA | NA |
| **Finland** | ✓ | ✓ | ✓ | 2.1 | 2 | 3.47 | 2.70 | 0.87 | 0.87 |
| **France** | ✓ | ✓ | ✓ | 0.9 | 5 | 2.45 | 2.60 | 0.75 | 0.73 |
| **Gabon** | ✓ | ✓ | ✓ | 4.7 | 2 | 1.82 | 1.24 | 0.46 | 0.39 |
| **Gambia** | ✓ | ✓ | ✓ | 4.3 | 2 | 1.20 | 0.93 | 0.37 | 0.49 |
| **Germany** | ✓ | ✓ | ✓ | 0.5 | 2 | 3.48 | 3.98 | 0.84 | 0.83 |
| **Ghana** | ✓ | ✓ | ✓ | 3.4 | 1 | 2.25 | -1.05 | 0.53 | 0.55 |
| **Greece** | ✓ | ✓ | ✓ | 3.6 | 4 | 1.96 | 1.64 | 0.55 | 0.61 |
| **Guatemala** | ✓ | ✓ | ✓ | 1.3 | 0 | 0.17 | -0.59 | 0.40 | 0.44 |
| **Guyana** | ✓ | ✓ | ✓ | 9.5 | 2 | 0.68 | 0.60 | 0.47 | 0.50 |
| **Honduras** | ✓ | ✓ | ✓ | 1.5 | 2 | -0.58 | -0.07 | 0.39 | 0.41 |
| **Hong Kong** |  | ✓ | ✓ | NA | 4 | -0.18 | 0.31 | 0.79 | 0.73 |
| **Hungary** | ✓ | ✓ | ✓ | 1.2 | 2 | -0.14 | 0.45 | 0.45 | 0.51 |
| **Iceland** | ✓ | ✓ | ✓ | 0.9 | 3 | 2.56 | 2.41 | NA | NA |
| **India** | ✓ | ✓ | ✓ | 3.9 | 4 | 0.29 | -1.38 | 0.48 | 0.49 |
| **Indonesia** |  | ✓ | ✓ | NA | 0 | 0.26 | -0.09 | 0.57 | 0.53 |
| **Iran** | ✓ | ✓ | ✓ | 0.8 | 2 | -0.15 | -1.05 | 0.44 | 0.39 |
| **Iraq** | ✓ | ✓ | ✓ | 1.8 | 2 | -0.19 | -0.91 | NA | NA |
| **Ireland** | ✓ | ✓ | ✓ | 1.1 | 4 | 3.02 | 2.52 | 0.82 | 0.81 |
| **Italy** | ✓ | ✓ | ✓ | 0.5 | 3 | 1.78 | 1.29 | 0.64 | 0.67 |
| **Jamaica** | ✓ | ✓ | ✓ | 0.7 | 3 | 1.36 | 1.92 | 0.55 | 0.57 |
| **Japan** | ✓ | ✓ | ✓ | 5.8 | 3 | 2.77 | 1.63 | 0.80 | 0.79 |
| **Jordan** | ✓ | ✓ | ✓ | 0.6 | 6 | 0.98 | 0.94 | 0.55 | 0.55 |
| **Kazakhstan** | ✓ | ✓ | ✓ | 3.0 | 5 | -0.31 | -0.73 | 0.52 | 0.53 |
| **Kenya** | ✓ | ✓ | ✓ | 2.9 | 2 | 1.35 | 1.07 | 0.45 | 0.46 |
| **Kiribati** |  | ✓ | ✓ | NA | 0 | NA | NA | NA | NA |
| **Kuwait** | ✓ | ✓ | ✓ | 0.5 | 5 | 1.15 | 0.38 | 0.66 | 0.58 |
| **Kyrgyzstan** | ✓ | ✓ | ✓ | 2.6 | 1 | 0.37 | 0.62 | 0.41 | 0.45 |
| **Lao PDR** | ✓ | ✓ | ✓ | 7.7 | 0 | -0.58 | -0.76 | NA | NA |
| **Latvia** | ✓ | ✓ | ✓ | 6.9 | 3 | 2.55 | 2.88 | 0.71 | 0.73 |
| **Lebanon** | ✓ | ✓ | ✓ | 0.6 | 1 | -0.07 | -1.16 | 0.44 | 0.45 |
| **Liberia** | ✓ | ✓ | ✓ | 5.1 | 2 | -0.04 | -0.03 | 0.40 | 0.44 |
| **Lithuania** | ✓ | ✓ | ✓ | 5.6 | 5 | 1.25 | 1.29 | 0.76 | 0.77 |
| **Luxembourg** | ✓ | ✓ | ✓ | 0.2 | 3 | 2.67 | 3.09 | 0.87 | 0.83 |
| **Macao** |  | ✓ | ✓ | NA | 3 | NA | NA | NA | NA |
| **Malawi** | ✓ | ✓ | ✓ | 4.3 | 3 | 2.23 | 1.20 | 0.46 | 0.52 |
| **Malaysia** | ✓ | ✓ | ✓ | 2.8 | 2 | -0.24 | -0.40 | 0.55 | 0.57 |
| **Maldives** | ✓ | ✓ | ✓ | 2.2 | 2 | 0.95 | -0.07 | NA | NA |
| **Mali** | ✓ | ✓ | ✓ | 9.1 | 2 | 1.01 | -0.69 | 0.47 | 0.40 |
| **Malta** | ✓ | ✓ | ✓ | 1.0 | 2 | 1.45 | 1.36 | 0.59 | 0.68 |
| **Marshall Islands** |  | ✓ | ✓ | NA | 0 | NA | NA | NA | NA |
| **Mauritius** | ✓ | ✓ | ✓ | 2.6 | 5 | 0.66 | -1.17 | 0.62 | 0.61 |
| **Mexico** | ✓ | ✓ | ✓ | 1.4 | 5 | -0.43 | -0.36 | 0.44 | 0.42 |
| **Micronesia** |  | ✓ | ✓ | NA | 0 | NA | NA | 0.48 | 0.56 |
| **Mongolia** | ✓ | ✓ | ✓ | 4.1 | 5 | 0.04 | 0.95 | 0.48 | 0.53 |
| **Montenegro** | ✓ | ✓ | ✓ | 1.1 | 5 | 1.14 | 1.35 | 0.48 | 0.56 |
| **Morocco** | ✓ | ✓ | ✓ | 1.1 | 5 | -0.26 | -0.61 | 0.50 | 0.48 |
| **Myanmar** | ✓ | ✓ | ✓ | 5.2 | 3 | -2.05 | -1.60 | 0.42 | 0.35 |
| **Namibia** | ✓ | ✓ | ✓ | 5.0 | 3 | 1.80 | 2.43 | 0.58 | 0.61 |
| **Nepal** | ✓ | ✓ | ✓ | 5.4 | 1 | 0.72 | 0.65 | 0.49 | 0.52 |
| **Netherlands** | ✓ | ✓ | ✓ | 0.5 | 4 | 2.00 | 2.21 | 0.85 | 0.83 |
| **New Zealand** | ✓ | ✓ | ✓ | 1.2 | 5 | 1.82 | 2.23 | 0.84 | 0.83 |
| **Niger** | ✓ | ✓ | ✓ | 11.3 | 3 | 1.70 | 0.95 | 0.47 | 0.44 |
| **Nigeria** | ✓ | ✓ | ✓ | 6.4 | 3 | -0.41 | -0.89 | 0.41 | 0.41 |
| **North Macedonia** | ✓ | ✓ | ✓ | 0.9 | 3 | 0.42 | -0.24 | 0.46 | 0.53 |
| **Norway** | ✓ | ✓ | ✓ | 1.4 | 3 | 2.37 | 2.37 | 0.88 | 0.89 |
| **Pakistan** | ✓ | ✓ | ✓ | 9.6 | 4 | -0.87 | -1.11 | 0.38 | 0.38 |
| **Palau** |  | ✓ | ✓ | NA | 0 | NA | NA | NA | NA |
| **Panama** | ✓ | ✓ | ✓ | 3.2 | 4 | 1.37 | 0.92 | 0.49 | 0.51 |
| **Papua New Guinea** | ✓ | ✓ | ✓ | 6.5 | 2 | -0.11 | -0.37 | NA | NA |
| **Paraguay** | ✓ |  | ✓ | 1.4 | 2 | 0.38 | 0.10 | 0.46 | 0.46 |
| **Peru** | ✓ |  | ✓ | 1.5 | 3 | 0.51 | 0.44 | 0.48 | 0.49 |
| **Philippines** | ✓ |  | ✓ | 5.9 | 4 | -0.71 | -0.74 | 0.47 | 0.46 |
| **Poland** | ✓ |  | ✓ | 1.3 | 2 | 0.44 | 0.87 | 0.63 | 0.64 |
| **Portugal** | ✓ |  | ✓ | 0.8 | 4 | 2.04 | 1.41 | 0.60 | 0.68 |
| **Qatar** | ✓ |  | ✓ | 1.4 | 2 | -1.05 | 1.08 | NA | NA |
| **Republic of Korea** | ✓ |  | ✓ | 1.0 | 5 | 2.25 | 2.18 | 0.74 | 0.74 |
| **Republic of Moldova** | ✓ |  | ✓ | 3.6 | 2 | 1.23 | 0.77 | 0.46 | 0.53 |
| **Romania** | ✓ |  | ✓ | 3.2 | 2 | 0.00 | 0.98 | 0.59 | 0.63 |
| **Russian Federation** | ✓ |  | ✓ | 3.0 | 4 | -0.96 | -0.81 | 0.46 | 0.44 |
| **Rwanda** | ✓ |  | ✓ | 5.9 | 5 | 0.51 | 0.29 | 0.60 | 0.63 |
| **Samoa** |  |  | ✓ | NA | 0 | NA | NA | NA | NA |
| **San Marino** | ✓ |  | ✓ | 0.0 | 0 | NA | NA | NA | NA |
| **Saudi Arabia** | ✓ |  | ✓ | 1.5 | 7 | -1.19 | -0.78 | NA | NA |
| **Senegal** | ✓ |  | ✓ | 4.6 | 4 | 1.73 | 0.96 | 0.56 | 0.55 |
| **Serbia** | ✓ |  | ✓ | 1.0 | 2 | 1.35 | 1.41 | 0.46 | 0.48 |
| **Seychelles** | ✓ |  | ✓ | 6.6 | 2 | 3.09 | 2.33 | NA | NA |
| **Sierra Leone** | ✓ |  | ✓ | 8.3 | 1 | 0.86 | 1.42 | 0.37 | 0.44 |
| **Singapore** |  |  | ✓ | NA | 0 | 2.53 | 3.13 | 0.86 | 0.78 |
| **Solomon Islands** | ✓ |  | ✓ | 11.4 | 2 | 0.83 | 0.33 | NA | NA |
| **Somalia** | ✓ |  | ✓ | 11.1 | 0 | -0.85 | -0.47 | NA | NA |
| **South Africa** | ✓ |  | ✓ | 4.4 | 2 | 1.06 | 0.77 | 0.52 | 0.57 |
| **South Sudan** | ✓ |  | ✓ | 7.0 | 1 | -1.80 | -2.80 | NA | NA |
| **Spain** | ✓ |  |  | 1.1 | 6 | 2.25 | 2.53 | 0.69 | 0.72 |
| **Sri Lanka** | ✓ |  |  | 3.3 | 4 | -0.15 | -0.99 | 0.48 | 0.50 |
| **Suriname** | ✓ |  |  | 4.5 | 2 | 0.55 | 0.21 | 0.46 | 0.49 |
| **Sweden** | ✓ |  |  | 0.9 | 2 | 4.04 | 3.60 | 0.83 | 0.85 |
| **Switzerland** | ✓ |  |  | 0.5 | 3 | 3.24 | 3.56 | NA | NA |
| **Tajikistan** | ✓ |  |  | 4.4 | 1 | -1.73 | -1.71 | NA | NA |
| **Thailand** | ✓ |  |  | 9.0 | 6 | -1.11 | -1.12 | 0.44 | 0.49 |
| **Timor-Leste** | ✓ |  |  | 7.8 | 3 | 0.92 | 1.16 | NA | NA |
| **Togo** | ✓ |  |  | 6.4 | 1 | 1.44 | 1.40 | 0.51 | 0.45 |
| **Tonga** | ✓ |  |  | 5.3 | 2 | NA | NA | NA | NA |
| **Tunisia** | ✓ |  |  | 0.7 | 4 | 0.36 | 1.00 | 0.50 | 0.52 |
| **Uganda** | ✓ |  |  | 4.4 | 2 | 0.12 | 0.12 | 0.43 | 0.39 |
| **United Kingdom of Great Britain and Northern Ireland** | ✓ |  |  | 0.4 | 4 | 2.02 | 2.50 | 0.79 | 0.78 |
| **United Republic of Tanzania** | ✓ |  |  | 3.9 | 4 | 1.19 | 1.25 | 0.44 | 0.47 |
| **United States of America** | ✓ |  |  | 1.3 | 7 | 2.56 | 2.07 | 0.72 | 0.70 |
| **Uruguay** | ✓ |  |  | 1.4 | 4 | 2.19 | 1.83 | 0.71 | 0.72 |
| **Uzbekistan** | ✓ |  |  | 3.4 | 0 | -0.02 | 0.22 | 0.45 | 0.50 |
| **Viet Nam** | ✓ |  |  | 7.8 | 5 | -0.62 | -0.63 | 0.44 | 0.49 |
| **Zambia** | ✓ |  |  | 4.9 | 2 | 0.71 | 0.11 | 0.43 | 0.45 |
| **Zimbabwe** | ✓ |  |  | 6.5 | 3 | -1.16 | -1.34 | 0.35 | 0.40 |
