# Supplementary material for: An analysis of global legislation and regulation related to drowning prevention
Source: PLOS Glob Public Health. 2026 Mar 25;6(3):e0005337. doi: 10.1371/journal.pgph.0005337 (PMC13016334; doi:10.1371/journal.pgph.0005337)
Supplement: S4 Table — (DOCX) [file pgph.0005337.s004.docx]

**Table S4. Regions represented in main models**

| World Bank regions by model | | | |
| --- | --- | --- | --- |
| **Model** | **Region** | **n** | **%** |
| M0 | Europe & Central Asia | 35 | 27.6% |
|  | Sub-Saharan Africa | 28 | 22.0% |
|  | East Asia & Pacific | 24 | 18.9% |
|  | Latin America & Caribbean | 20 | 15.7% |
|  | Middle East & North Africa | 10 | 7.9% |
|  | South Asia | 6 | 4.7% |
|  | North America | 2 | 1.6% |
|  | Unknown | 2 | 1.6% |
| M1 | Europe & Central Asia | 28 | 26.9% |
|  | Sub-Saharan Africa | 21 | 20.2% |
|  | East Asia & Pacific | 19 | 18.3% |
|  | Latin America & Caribbean | 18 | 17.3% |
|  | Middle East & North Africa | 8 | 7.7% |
|  | South Asia | 6 | 5.8% |
|  | North America | 2 | 1.9% |
|  | Unknown | 2 | 1.9% |
| M2 | Europe & Central Asia | 35 | 27.6% |
|  | Sub-Saharan Africa | 28 | 22.0% |
|  | East Asia & Pacific | 24 | 18.9% |
|  | Latin America & Caribbean | 20 | 15.7% |
|  | Middle East & North Africa | 10 | 7.9% |
|  | South Asia | 6 | 4.7% |
|  | North America | 2 | 1.6% |
|  | Unknown | 2 | 1.6% |
