# Supplementary material for: An analysis of global legislation and regulation related to drowning prevention
Source: PLOS Glob Public Health. 2026 Mar 25;6(3):e0005337. doi: 10.1371/journal.pgph.0005337 (PMC13016334; doi:10.1371/journal.pgph.0005337)
Supplement: S5 Table — (DOCX) [file pgph.0005337.s005.docx]

**Table S5. Summary of regional models**

|  | **M3** | **M4** | **M5** |
| --- | --- | --- | --- |
| GDP | 0.477 | 0.468 | 0.523 |
|  | (0.465) | (0.499) | (0.697) |
| Avg temp | 1.099 | 1.057 | 1.034 |
|  | (0.330) | (0.328) | (0.530) |
| Legislative enforcement | 0.937 | 0.920 | 0.879 |
|  | (0.567) | (0.561) | (0.694) |
| Alcohol | 1.091 | 1.071 | 1.069 |
|  | (0.305) | (0.291) | (0.503) |
| Health-sector capacity | 1.134 | 1.129 | 1.110 |
|  | (0.497) | (0.479) | (0.774) |
| Urbanisation | 0.845 | 0.849 | 0.812 |
|  | (0.455) | (0.431) | (0.733) |
| Disaster exposure | 1.041 | 1.077 | 1.113 |
|  | (0.432) | (0.451) | (0.590) |
| Water & sanitation | 0.833 | 0.840 | 0.801 |
|  | (0.249) | (0.174) | (0.559) |
| Public-health spend | 1.214 | 1.281 | 1.277 |
|  | (1.574) | (1.785) | (1.943) |
| Total laws | 1.001 |  | 0.975 |
|  | (0.064) |  | (0.136) |
| Enforcement strength |  | 1.001 | 1.005 |
|  |  | (0.006) | (0.048) |
| Total laws × enforcement |  |  | 1.000 |
|  |  |  | (0.003) |
| Num.Obs. | 19 | 19 | 19 |
| RMSE | 10121.34 | 8217.38 | 5067.25 |
| Std.Errors | M3 | M4 | M5 |

+ p < 0.1, * p < 0.05, ** p < 0.01, *** p < 0.001
