# Supplementary material for: An analysis of global legislation and regulation related to drowning prevention
Source: PLOS Glob Public Health. 2026 Mar 25;6(3):e0005337. doi: 10.1371/journal.pgph.0005337 (PMC13016334; doi:10.1371/journal.pgph.0005337)
Supplement: S6 Table — NB2 vs PPML. (DOCX) [file pgph.0005337.s006.docx]

**Table S6. Robustness. NB2 vs PPML**

|  | **NB2 M0** | **NB2 M1** | **NB2 M2** | **PPML M0** | **PPML M1** | **PPML M2** |
| --- | --- | --- | --- | --- | --- | --- |
| GDP | 0.934 | 0.854 | 0.918 | 0.798 | 0.659 | 0.860 |
|  | (0.180) | (0.244) | (0.182) | (0.418) | (0.275) | (0.357) |
| Avg temp | 1.044 | 1.087 | 1.040 | 0.895 | 1.220 | 0.940 |
|  | (0.089) | (0.097) | (0.088) | (0.224) | (0.260) | (0.194) |
| Legislative enforcement | 0.971 | 0.963 | 0.985 | 0.852 | 0.970 | 0.895 |
|  | (0.108) | (0.162) | (0.111) | (0.177) | (0.212) | (0.158) |
| Alcohol | 1.236* | 1.315+ | 1.240* | 0.988 | 1.171 | 0.970 |
|  | (0.113) | (0.193) | (0.116) | (0.212) | (0.245) | (0.199) |
| Health-sector capacity | 1.005 | 1.054 | 0.978 | 0.838 | 1.155 | 0.695 |
|  | (0.114) | (0.158) | (0.113) | (0.200) | (0.382) | (0.166) |
| Urbanisation | 1.220 | 0.987 | 1.252 | 1.088 | 0.985 | 1.114 |
|  | (0.179) | (0.215) | (0.189) | (0.308) | (0.409) | (0.291) |
| Disaster exposure | 1.104 | 1.134 | 1.109 | 1.103 | 1.030 | 1.164 |
|  | (0.118) | (0.165) | (0.122) | (0.246) | (0.285) | (0.221) |
| Water & sanitation | 0.809* | 0.791 | 0.811* | 0.975 | 0.804 | 0.854 |
|  | (0.087) | (0.113) | (0.085) | (0.389) | (0.285) | (0.244) |
| Public-health spend | 0.808 | 0.826 | 0.823 | 1.094 | 1.190 | 1.248 |
|  | (0.148) | (0.205) | (0.152) | (0.493) | (0.522) | (0.458) |
| National strategy |  | 1.034 |  |  | 0.698 |  |
|  |  | (0.279) |  |  | (0.261) |  |
| Disaster policy |  | 1.047 |  |  | 2.038* |  |
|  |  | (0.354) |  |  | (0.666) |  |
| Private-pool fencing |  | 0.485 |  |  | 0.274 |  |
|  |  | (0.326) |  |  | (0.237) |  |
| Public-pool fencing |  | 0.702 |  |  | 0.526 |  |
|  |  | (0.253) |  |  | (0.234) |  |
| Water-transport safety |  | 0.621 |  |  | 0.801 |  |
|  |  | (0.218) |  |  | (0.388) |  |
| Lifejacket requirement |  | 1.637 |  |  | 2.077 |  |
|  |  | (0.507) |  |  | (1.389) |  |
| Alcohol regulation near water |  | 1.204 |  |  | 1.511 |  |
|  |  | (0.340) |  |  | (0.473) |  |
| Total laws (global) |  |  | 1.053 |  |  | 1.198 |
|  |  |  | (0.051) |  |  | (0.144) |
| Num.Obs. | 89 | 71 | 89 | 89 | 71 | 89 |
| RMSE | 2713.96 | 2690.32 | 2780.76 | 1307.17 | 509.28 | 996.14 |
| Std.Errors | NB2 M0 | NB2 M1 | NB2 M2 | PPML M0 | PPML M1 | PPML M2 |

+ p < 0.1, * p < 0.05, ** p < 0.01, *** p < 0.001
