# Supplementary material for: An analysis of global legislation and regulation related to drowning prevention
Source: PLOS Glob Public Health. 2026 Mar 25;6(3):e0005337. doi: 10.1371/journal.pgph.0005337 (PMC13016334; doi:10.1371/journal.pgph.0005337)
Supplement: S7 Table — Influence-trimmed NB2 (drop top 5% Cook’s D). (DOCX) [file pgph.0005337.s007.docx]

**Table S7. Robustness. Influence-trimmed NB2 (drop top 5% Cook's D)**

|  | **NB2 (trim) M0** | **NB2 (trim) M1** | **NB2 (trim) M2** |
| --- | --- | --- | --- |
| GDP | 1.048 | 1.012 | 1.035 |
|  | (0.169) | (0.214) | (0.167) |
| Avg temp | 1.073 | 1.105 | 1.068 |
|  | (0.088) | (0.094) | (0.089) |
| V-DEM enforcement | 0.923 | 0.917 | 0.929 |
|  | (0.070) | (0.076) | (0.071) |
| Alcohol | 1.298** | 1.402*** | 1.297** |
|  | (0.105) | (0.133) | (0.104) |
| Health-sector robustness | 1.056 | 1.038 | 1.042 |
|  | (0.109) | (0.115) | (0.110) |
| Urbanisation | 1.335** | 1.059 | 1.357** |
|  | (0.133) | (0.129) | (0.137) |
| Disaster exposure | 1.000 | 1.001 | 1.003 |
|  | (0.074) | (0.088) | (0.074) |
| Water & sanitation | 0.743* | 0.759+ | 0.747* |
|  | (0.096) | (0.113) | (0.097) |
| Public-health spend | 0.700*** | 0.666*** | 0.707*** |
|  | (0.072) | (0.082) | (0.073) |
| National strategy |  | 1.237 |  |
|  |  | (0.198) |  |
| Disaster policy |  | 0.772+ |  |
|  |  | (0.113) |  |
| Private-pool fencing |  | 0.582 |  |
|  |  | (0.198) |  |
| Public-pool fencing |  | 0.806 |  |
|  |  | (0.153) |  |
| Water-transport safety |  | 0.602+ |  |
|  |  | (0.159) |  |
| Lifejacket requirement |  | 1.386 |  |
|  |  | (0.280) |  |
| Alcohol regulation near water |  | 1.221 |  |
|  |  | (0.195) |  |
| Total laws (global) |  |  | 1.031 |
|  |  |  | (0.046) |
| Num.Obs. | 84 | 67 | 84 |
| RMSE | 536.56 | 3014.52 | 548.91 |

+ p < 0.1, * p < 0.05, ** p < 0.01, *** p < 0.001
