# Supplementary material for: An analysis of global legislation and regulation related to drowning prevention
Source: PLOS Glob Public Health. 2026 Mar 25;6(3):e0005337. doi: 10.1371/journal.pgph.0005337 (PMC13016334; doi:10.1371/journal.pgph.0005337)
Supplement: S8 Table — (DOCX) [file pgph.0005337.s008.docx]

**Table S8. Countries excluded from influence trimmed NB2**

| **Model** | **N dropped** | **Dropped** |
| --- | --- | --- |
| M0 | 5 | China, Ethiopia, India, Japan, Pakistan |
| M1 | 4 | China, Ethiopia, Japan, Pakistan |
| M2 | 5 | China, Ethiopia, India, Japan, Pakistan |
