# Supplementary material for: An analysis of global legislation and regulation related to drowning prevention
Source: PLOS Glob Public Health. 2026 Mar 25;6(3):e0005337. doi: 10.1371/journal.pgph.0005337 (PMC13016334; doi:10.1371/journal.pgph.0005337)
Supplement: S9 Table — (DOCX) [file pgph.0005337.s009.docx]

**Table S9. Dimension reduction**

|  | **PCA→NB2/PPML** |
| --- | --- |
| (Intercept) | 0.000*** |
|  | (0.000) |
| National strategy | 1.034 |
|  | (0.212) |
| Disaster policy | 1.153 |
|  | (0.255) |
| Private-pool fencing | 0.590+ |
|  | (0.159) |
| Public-pool fencing | 1.172 |
|  | (0.315) |
| Water-transport safety | 0.791 |
|  | (0.146) |
| Lifejacket requirement | 1.202 |
|  | (0.229) |
| Alcohol regulation near water | 1.139 |
|  | (0.165) |
| PC1 | 0.778*** |
|  | (0.032) |
| PC2 | 0.829** |
|  | (0.059) |
| PC3 | 0.857* |
|  | (0.063) |
| PC4 | 1.006 |
|  | (0.115) |
| Num.Obs. | 104 |
| RMSE | 2007.39 |
| Std.Errors | Custom |

+ p < 0.1, * p < 0.05, ** p < 0.01, *** p < 0.001
