# Supplementary material for: An analysis of global legislation and regulation related to drowning prevention
Source: PLOS Glob Public Health. 2026 Mar 25;6(3):e0005337. doi: 10.1371/journal.pgph.0005337 (PMC13016334; doi:10.1371/journal.pgph.0005337)
Supplement: S10 Table — (DOCX) [file pgph.0005337.s010.docx]

**Table S10. Diagnostics**

| **Model** | **N** | **k** | **Max VIF** | **Disp p** | **Zero p** | **Unif p** | **Moran I** | **Moran p** |
| --- | --- | --- | --- | --- | --- | --- | --- | --- |
| M0 | 127 | 9 | 8.351 | 0.508 | 1 | 0.654 | -0.032 | 0.625 |
| M2 | 127 | 10 | 8.378 | 0.473 | 1 | 0.668 | -0.032 | 0.628 |
| M1 | 104 | 16 | 12.416 | 0.377 | 1 | 0.923 | -0.034 | 0.647 |
