# Supplementary material for: An analysis of global legislation and regulation related to drowning prevention
Source: PLOS Glob Public Health. 2026 Mar 25;6(3):e0005337. doi: 10.1371/journal.pgph.0005337 (PMC13016334; doi:10.1371/journal.pgph.0005337)
Supplement: S11 Table — (DOCX) [file pgph.0005337.s011.docx]

**Table S11. Random forest permutation importance**

| **Variable** | **Mean importance** | **95% CI** |
| --- | --- | --- |
| Public-health spend | 0.269 | [0.167, 0.422] |
| Water & sanitation | 0.245 | [0.155, 0.366] |
| GDP | 0.237 | [0.144, 0.361] |
| Urbanisation | 0.226 | [0.111, 0.354] |
| Avg temp | 0.113 | [0.061, 0.204] |
| Health-sector robustness | 0.096 | [0.059, 0.146] |
| Legislative enforcement | 0.091 | [0.048, 0.163] |
| Alcohol | 0.077 | [0.035, 0.131] |
| Disaster exposure | 0.035 | [0.017, 0.066] |
| National strategy | 0.020 | [0.009, 0.041] |
| Alcohol near water regulation | 0.011 | [0.003, 0.030] |
| Disaster policy | 0.010 | [0.003, 0.028] |
| Water-transport safety | 0.009 | [0.001, 0.028] |
| Public-pool fencing | 0.008 | [0.001, 0.024] |
| Lifejacket requirement | 0.004 | [0.000, 0.011] |
| Private-pool fencing | 0.003 | [0.000, 0.010] |
