# Supplementary material for: An analysis of global legislation and regulation related to drowning prevention
Source: PLOS Glob Public Health. 2026 Mar 25;6(3):e0005337. doi: 10.1371/journal.pgph.0005337 (PMC13016334; doi:10.1371/journal.pgph.0005337)
Supplement: S12 Table — (DOCX) [file pgph.0005337.s012.docx]

**Table S12. Interaction between enforcement capacity × law**

| **Legislative Variable** | **IRR (with potential mediators)** | **IRR (without potential mediators)** | **% Change** | **Interpretation** |
| --- | --- | --- | --- | --- |
| **National strategy** | 1.044 | 1.124 | **7.6%** | Minimal change |
| **Disaster policy** | 1.104 | 1.045 | **5.3%** | Minimal change |
| **Private-pool fencing** | 0.638 | 0.625 | **2.0%** | Minimal change |
| **Public-pool fencing** | 1.039 | 0.989 | **4.8%** | Minimal change |
| **Water-transport safety** | 0.759 | 0.723 | **4.6%** | Minimal change |
| **Lifejacket requirement** | 1.279 | 1.399 | **9.4%** | Minimal change |
| **Alcohol regulation** | 1.163 | 1.180 | **1.5%** | Minimal change |
| **Total laws (global count)** | 1.032 | 1.018 | **1.3%** | Minimal change |
