# Supplementary figures and images for: An analysis of global legislation and regulation related to drowning prevention
Source: PLOS Glob Public Health. 2026 Mar 25;6(3):e0005337. doi: 10.1371/journal.pgph.0005337 (PMC13016334; doi:10.1371/journal.pgph.0005337)

**Figure S1. Summary of regional models**


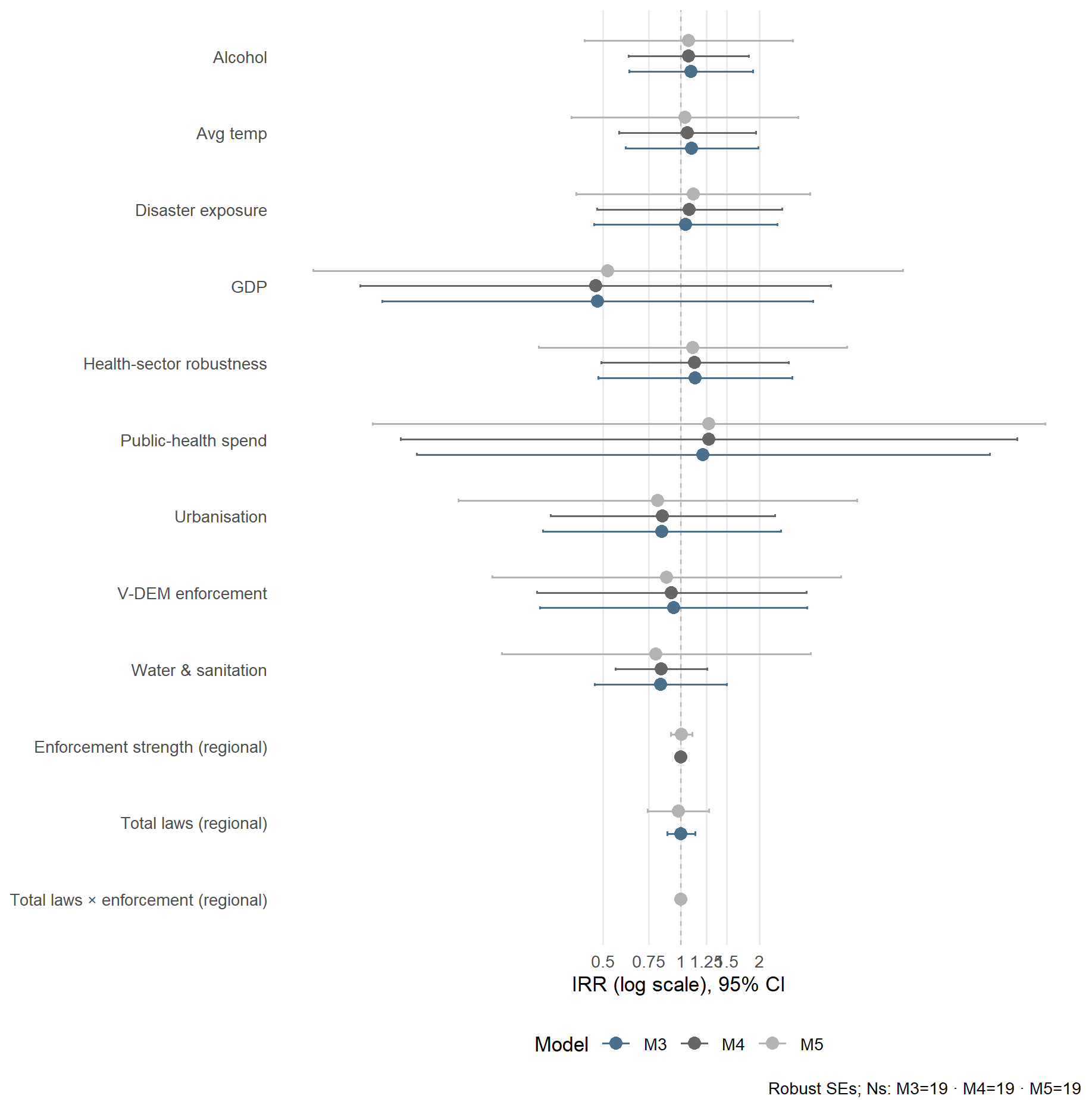

Supplement: S1 Fig — (DOCX) [file pgph.0005337.s013.docx]

**Figure S2**

**
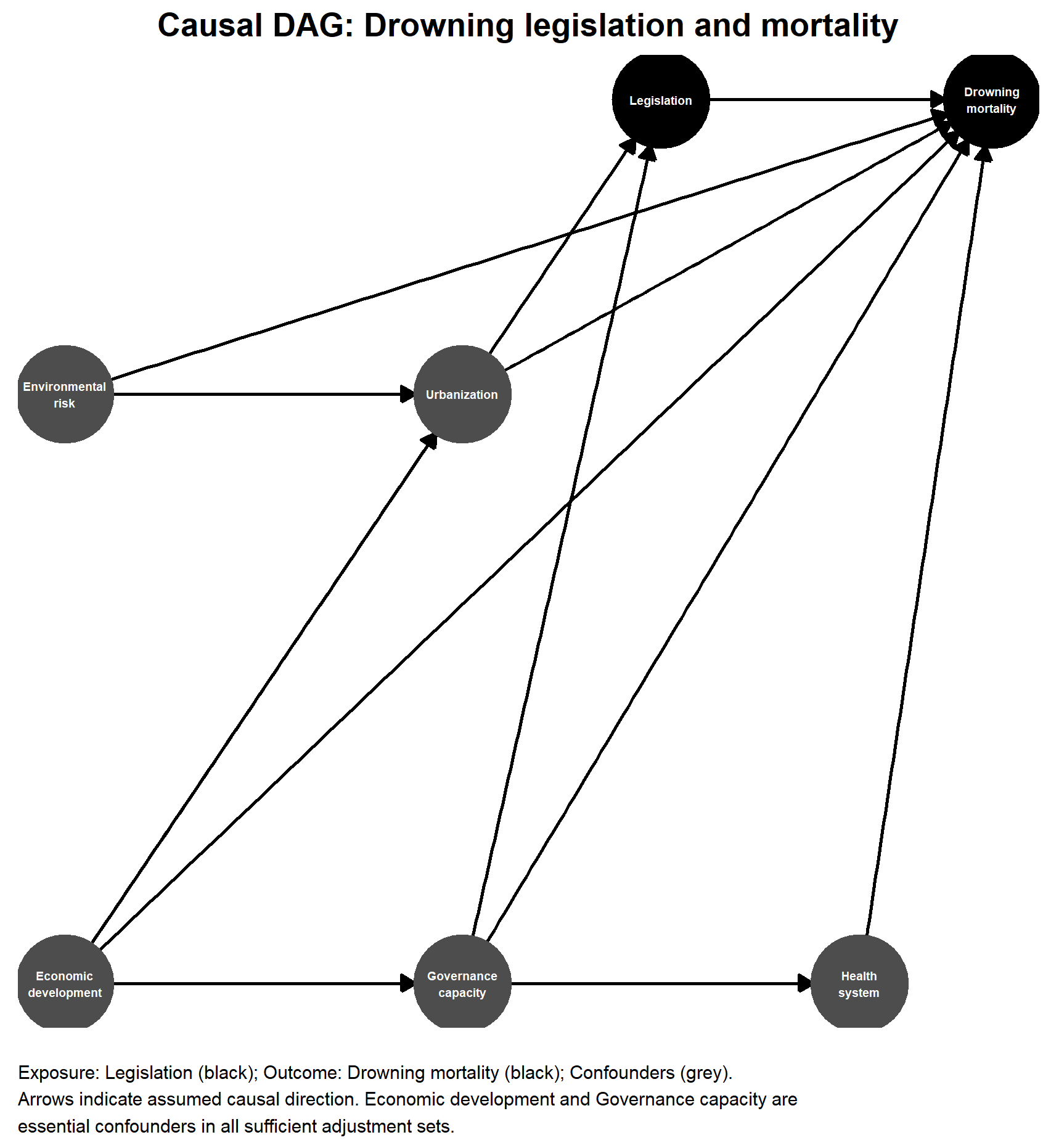
**

Supplement: S2 Fig — (DOCX) [file pgph.0005337.s014.docx]
