# Supplementary material for: An analysis of global legislation and regulation related to drowning prevention
Source: PLOS Glob Public Health. 2026 Mar 25;6(3):e0005337. doi: 10.1371/journal.pgph.0005337 (PMC13016334; doi:10.1371/journal.pgph.0005337)
Supplement: Attachment — Submitted filename: Reviewer Report_Ryan Essex.docx [file pgph.0005337.s015.docx]

**Reviewer Report**

This is an ambitious and timely article assessing the association between drowning-related legislation and mortality across countries. The topic is of clear global health relevance, and the authors’ effort to compile a cross-national dataset and triangulate multiple modelling approaches is commendable. The paper is well-written, transparent about methods, and presents extensive supplementary analyses. However, there are important methodological and interpretive issues that weaken the strength of the conclusions. In particular, sample size limitations, potential overfitting, choice of outcome modelling, and lack of clarity around causal interpretation require careful attention before publication.

**Abstract**

- Strengths: Clear statement of objectives and methods; multiple models (OLS, GLM, RF) reported; key findings succinctly conveyed.
- Concerns:
  - Wording overstates null results (e.g., “laws do not matter”); should instead emphasize “no consistent association observed at country level.”
  - Statistical detail is limited include effect sizes and 95% CIs for at least one key predictor to give context.
  - Policy relevance is implied but not explicitly linked to the observed dominance of structural determinants.
  - if Journal permits then add key words from MESH

**Introduction**

- Strengths: Good framing of drowning as a global public health issue and rationale for examining legislation.
- Concerns:
  - Literature review is skewed towards descriptive burden; limited discussion of prior evaluations of policy effectiveness (e.g., natural experiments, case studies).
  - Causal pathways (how laws may reduce drowning through enforcement, infrastructure, or behaviour change) need clearer articulation.
  - The introduction should foreshadow limitations of ecological cross-country comparisons (endogeneity, measurement error).

**Methods**

- Strengths: Transparent description of data sources; multiple model specifications attempted; inclusion of robustness checks.
- Concerns:
  1. Outcome modelling: OLS on rates is suboptimal; drowning deaths are count data → negative binomial or Poisson with population offset would be more appropriate. Gamma GLM is not well justified.
  2. Covariate selection: Many structural predictors included simultaneously despite ~100 observations; risks overfitting. Consider penalized regression or dimension reduction (e.g., PCA).
  3. Regional models (n=19): Severely underpowered for multiple predictors and interactions; these should be presented as exploratory only.
  4. Law measurement: Binary presence/absence recoding risks attenuation bias; more nuanced scoring of comprehensiveness or enforcement would be preferable.
  5. Causality: Cross-sectional design cannot address temporality; countries may adopt laws in response to drowning burden. This limitation should be made explicit.
  6. Diagnostics: No evidence provided of checking residuals, multicollinearity (VIFs), or spatial autocorrelation. These are essential for ecological analyses.

**Results**

- Strengths: Clearly presented tables; coefficients and CIs reported; random forest adds triangulation.
- Concerns:
  1. Interpretation: Emphasis on null law effects is too strong given wide CIs and measurement limitations.
  2. Sample size: Regional models produce negative adjusted R²; interpret cautiously or drop from main text.
  3. Robustness checks: Outlier exclusion changes significance of key covariates, suggesting instability. These shifts should be highlighted.
  4. Consistency: Urbanisation, alcohol, and sanitation repeatedly emerge as significant predictors, this stability deserves more emphasis.
  5. Interactions: Law × enforcement interactions have huge SEs and are uninterpretable at n=19; presenting them risks misleading readers.
  6. Models 4-6 (n=19): The results of these models should be interpreted with extreme caution. With 10+ parameters and n=19, the models are severely overfit (as indicated by negative adjusted R² values). The coefficients are unstable and unreliable. These results should be de-emphasized in the main text and moved to the supplement with a strong caveat.

**Discussion**

- Strengths: Honest acknowledgment of some limitations; appropriate recognition of structural determinants.
- Concerns:
  1. Overgeneralisation — concluding that “laws do not reduce drowning” is not supported; better phrasing: “we did not detect systematic associations at country level.”
  2. Limited comparison to case-based or quasi-experimental evidence showing effectiveness of pool fencing or boating regulations.
  3. Policy recommendations are vague; should connect observed results (structural determinants dominate) to integrated strategies combining legislation with infrastructure and enforcement.
  4. Small sample and measurement issues are mentioned but downplayed; they deserve central placement.
  5. Reframing Null Results: The authors appropriately avoid overstating the null findings, correctly pointing to data limitations and the potential for legislation to be effective in specific contexts. They could strengthen this by more explicitly stating that the study finds no evidence of an association at the ecological level, rather than stating legislation is ineffective. The distinction is important.
  6. Limitations: The limitations section is good but should be more forceful on two points: 1) The ecological fallacy should be named and discussed as a primary limitation. 2) The severe power issue with the regional models (n=19) must be highlighted as a major constraint.

**Tables and Figures**

- Strengths: Informative and mostly well formatted.
- Concerns:
  - Forest plots or standardized coefficients would aid comparability of effect sizes.
  - Tables with >10 predictors for n=19 regions are not meaningful; consider moving to appendix.
  - Random forest importance plots should include uncertainty estimates (bootstrapped importance).

**Supplementary Material**

- Strengths: Very transparent, showing multiple specifications, data sources, and robustness checks.
- Concerns:
  - Gamma GLM results difficult to interpret; justify inclusion or simplify.
  - Outlier-exclusion results should specify which countries were dropped.
  - Regional analyses should be explicitly marked exploratory.

The article addresses a relevant question and shows commendable transparency. However, methodological and interpretive revisions are required:

1. Reanalyse with count-based models (negative binomial with population offset).
2. Downplay or remove unstable regional/interaction models.
3. Clarify measurement limitations of law variables.
4. Reframe conclusions to avoid overstatement and highlight structural determinants.
5. Expand on diagnostics (collinearity, spatial autocorrelation, influence analysis).
