# Supplementary material for: An analysis of global legislation and regulation related to drowning prevention
Source: PLOS Glob Public Health. 2026 Mar 25;6(3):e0005337. doi: 10.1371/journal.pgph.0005337 (PMC13016334; doi:10.1371/journal.pgph.0005337)
Supplement: Attachment — Submitted filename: PGPH Review.docx [file pgph.0005337.s016.docx]

This study analyzes global legislation and regulation related to drowning prevention.

Please see my comments below.

Abstract

A scientific study should be replicable. In the abstract the study type is unclear. Insufficient methodological details are given to the reader to understand what and how it was done?

Introduction

The introduction is written in an op-ed style. Most statements are without references. The literature search and knowledge gap are not clearly defined.

Novelty

This data is utilized from WHO report. What new information is it adding to the literature?

Methods

Insufficient details regarding the 127 countries so we cannot understand who is included in this analysis. Grouping and interpreting high-Income countries and low-income countries together statistically is not the right approach to deduce meaningful results.

Results and Discussion

The findings identify known predictors of drowning, with negative results for legislative measures, ultimately not revealing anything meaningful and novel to add to the literature
