# Supplementary material for: An analysis of global legislation and regulation related to drowning prevention
Source: PLOS Glob Public Health. 2026 Mar 25;6(3):e0005337. doi: 10.1371/journal.pgph.0005337 (PMC13016334; doi:10.1371/journal.pgph.0005337)
Supplement: Attachment — Submitted filename: Response.docx [file pgph.0005337.s018.docx]

| **Comment** | **Response** | **Location** |
| --- | --- | --- |
| **Editor** |  |  |
| This paper is very detailed, important, and novel use of global data to analyse drowning mortality and its association with legislation. The contribution is significant, as there has been little comparable work at this scale. That said, a few areas could be clarified and strengthened. The methods would benefit from greater detail on the specific countries included and on any exclusions made, so that readers have a clearer sense of the study population. The list of covariates is extensive, but at times gives the impression that most available variables were included rather than only those with strong evidence for relevance; more justification for their selection would be helpful, and the appendix should ideally avoid listing variables that were not ultimately analysed. The methods and results, while rigorous, could also be written in a way that is slightly easier to follow. Finally, while I recognize the constraints of global data availability, some important factors such as swimming and CPR skills, emergency rescue services, and early warning systems are missing from the analysis. Early warning system coverage in particular may be available through other global datasets, and could be considered for inclusion. At minimum, the absence of such variables should be acknowledged more explicitly. | Thank you very much for your feedback.  We have taken on your feedback and the feedback from both reviewers and made substantive changes to the paper, which we think makes it far stronger and more robust.  In terms of your specific requests, we have included several further supplementary tables that provide greater information in regards to our methods and data. In this revision, we have expanded on our rationale to utilised a LASSO approach in variable selection, you will also see we have included analyses that utilise PCA, to reduce the number of covariates in our models – we hope this addresses this issue. We have also included a list of variables – including those which did not make it into final modelling as supplementary material.  You will see we have made substantive changes to the results and methods section, so we now hope things are a little clearer.  In terms of these other factors, like CPR and swimming skills, we would have loved to include these variables, but could not find any reliable data (at least for a substantive number of countries). Our analysis of course cannot account for these factors and we do acknowledge these limitations re. confounding and data limitations in our discussion section. | Methods/  Results/  Limitations/  Supp. material |
| **Reviewer 1** |  |  |
| This is an ambitious and timely article assessing the association between drowning-related legislation and mortality across countries. The topic is of clear global health relevance, and the authors’ effort to compile a cross-national dataset and triangulate multiple modelling approaches is commendable. The paper is well-written, transparent about methods, and presents extensive supplementary analyses. However, there are important methodological and interpretive issues that weaken the strength of the conclusions. In particular, sample size limitations, potential overfitting, choice of outcome modelling, and lack of clarity around causal interpretation require careful attention before publication. | Thank you very much for your kind and helpful feedback. We largely agree with all of your suggestions and have attempted to implement them all. As you will see we have made substantive changes to the methods, results and discussion section based around updated negative binomial modelling. We hope we have adjusted our language appropriately as well, but if we have missed anything, we would be happy to work with you on any further revisions. |  |
| **Abstract**   - Strengths: Clear statement of objectives and methods; multiple models (OLS, GLM, RF) reported; key findings succinctly conveyed. - Concerns:   - Wording overstates null results (e.g., “laws do not matter”); should instead emphasize “no consistent association observed at country level.”   - Statistical detail is limited include effect sizes and 95% CIs for at least one key predictor to give context.   - Policy relevance is implied but not explicitly linked to the observed dominance of structural determinants.   - if Journal permits then add key words from MESH | Thank you  We have updated phrasing throughout to be more cautious re. “laws do not matter”. Our statements should now align better with the more balanced and appropriate “no associated with observed at country level” phrasing. We have done this in the abstract and throughout the paper.  We have included some sample effect sizes and CIs  We have been more explicit in terms of policy and recommendations that could be taken away from our findings.  We have added keywords in our submission, we believe these are MESH, but would be happy to work with the editors on this if we are mistaken. | Abstract |
| **Introduction**   - Strengths: Good framing of drowning as a global public health issue and rationale for examining legislation. - Concerns:   - Literature review is skewed towards descriptive burden; limited discussion of prior evaluations of policy effectiveness (e.g., natural experiments, case studies).   - Causal pathways (how laws may reduce drowning through enforcement, infrastructure, or behaviour change) need clearer articulation.   - The introduction should foreshadow limitations of ecological cross-country comparisons (endogeneity, measurement error). | We have expanded our introduction and introduced some more literature in this space. Sadly, there just isn’t a great deal and only a handful of countries that have attempted larger designs (i.e. studies that were not case studies). We have alluded to some causal pathways, but are fairly cautious here as there is a paucity on evidence here as well.  More generally, you will see we have made substantive changes to the introduction, we hope that these better set the paper up and foreshadow its limitations. We are also more explicit about this immediately after stating our research questions. | Introduction |
| **Methods**   - Strengths: Transparent description of data sources; multiple model specifications attempted; inclusion of robustness checks. - Concerns:   1. Outcome modelling: OLS on rates is suboptimal; drowning deaths are count data → negative binomial or Poisson with population offset would be more appropriate. Gamma GLM is not well justified.   2. Covariate selection: Many structural predictors included simultaneously despite ~100 observations; risks overfitting. Consider penalized regression or dimension reduction (e.g., PCA).   3. Regional models (n=19): Severely underpowered for multiple predictors and interactions; these should be presented as exploratory only.   4. Law measurement: Binary presence/absence recoding risks attenuation bias; more nuanced scoring of comprehensiveness or enforcement would be preferable.   5. Causality: Cross-sectional design cannot address temporality; countries may adopt laws in response to drowning burden. This limitation should be made explicit.   6. Diagnostics: No evidence provided of checking residuals, multicollinearity (VIFs), or spatial autocorrelation. These are essential for ecological analyses. | We have made substantive changes to our methods. The major changes we have made align with your suggestion, we have primarily relied on negative binomial modelling and we have moved the regional analysis to our supplementary material – we completely agree with your feedback here. In terms of each point   1. We have now switched to negative binomial models (we tried Poisson, but had overdispersion). We have dropped Gamma, OLS etc models 2. We acknowledge this risk. We have updated our robustness strategy in line with our updated modelling. Included here is a strategy that implements PCA – reducing these covariates to 4 and leaving our core findings largely intact. 3. Regional models have been dropped to supplementary material – we do not put any weight on them in the discuss and advise caution in their interpretation. 4. We did consider this, however could not do it with the data available – we have noted this as a limitation. 5. We address this in our limitations and more generally are more forceful on a number of points here 6. We ran diagnostics on our updated models and included the results as supplementary material | Methods |
| **Results**   - Strengths: Clearly presented tables; coefficients and CIs reported; random forest adds triangulation. - Concerns:   1. Interpretation: Emphasis on null law effects is too strong given wide CIs and measurement limitations.   2. Sample size: Regional models produce negative adjusted R²; interpret cautiously or drop from main text.   3. Robustness checks: Outlier exclusion changes significance of key covariates, suggesting instability. These shifts should be highlighted.   4. Consistency: Urbanisation, alcohol, and sanitation repeatedly emerge as significant predictors, this stability deserves more emphasis.   5. Interactions: Law × enforcement interactions have huge SEs and are uninterpretable at n=19; presenting them risks misleading readers.   6. Models 4-6 (n=19): The results of these models should be interpreted with extreme caution. With 10+ parameters and n=19, the models are severely overfit (as indicated by negative adjusted R² values). The coefficients are unstable and unreliable. These results should be de-emphasized in the main text and moved to the supplement with a strong caveat. | 1. We hope we offer a more balanced approach in our language here and hope dropping regional models to supplementary material helps with this concern 2. Dropped 3. We have updated our strategy re. robustness to align with our new approach to modelling. We have noted where things tighten and where instability exists – we welcome any further feedback however 4. We hope we have emphasised this in our discussion and results 5. We have dropped regional models from our interaction analysis. The interaction models are only based on global data and have n = 88 – 104 (this has been made clearer) 6. Again, these have been dropped and we have advised caution in their interpretation | Results |
| **Discussion**   - Strengths: Honest acknowledgment of some limitations; appropriate recognition of structural determinants. - Concerns:   1. Overgeneralisation — concluding that “laws do not reduce drowning” is not supported; better phrasing: “we did not detect systematic associations at country level.”   2. Limited comparison to case-based or quasi-experimental evidence showing effectiveness of pool fencing or boating regulations.   3. Policy recommendations are vague; should connect observed results (structural determinants dominate) to integrated strategies combining legislation with infrastructure and enforcement.   4. Small sample and measurement issues are mentioned but downplayed; they deserve central placement.   5. Reframing Null Results: The authors appropriately avoid overstating the null findings, correctly pointing to data limitations and the potential for legislation to be effective in specific contexts. They could strengthen this by more explicitly stating that the study finds no evidence of an association at the ecological level, rather than stating legislation is ineffective. The distinction is important.   6. Limitations: The limitations section is good but should be more forceful on two points: 1) The ecological fallacy should be named and discussed as a primary limitation. 2) The severe power issue with the regional models (n=19) must be highlighted as a major constraint. | More generally we hope we offer a more balanced and forthright discussion when it comes to our findings and the studies limitations   1. We have now addressed this language 2. We have added some of the other literature in this space, however as we noted above, studies related to the effectiveness of legislation/regulation in drowning prevention are limited 3. We are more explicit with our recommendations when it comes to policy and what can be taken away from this study. 4. As noted above, we have dropped the regional models to supplementary material and been more explicit with this, so hope this is addressed. 5. We have been more explicit with this 6. We have also been more forthright here. | Discussion |
| **Tables and Figures**   - Strengths: Informative and mostly well formatted. - Concerns:   - Forest plots or standardized coefficients would aid comparability of effect sizes.   - Tables with >10 predictors for n=19 regions are not meaningful; consider moving to appendix.   - Random forest importance plots should include uncertainty estimates (bootstrapped importance). | Thank you – we have included forest plots in the main text and moved regional analyses to supplementary material. We have now included bootstrapped CIs for our random forest analysis. | Tables and figures |
| **Supplementary Material**   - Strengths: Very transparent, showing multiple specifications, data sources, and robustness checks. - Concerns:   - Gamma GLM results difficult to interpret; justify inclusion or simplify.   - Outlier-exclusion results should specify which countries were dropped.   - Regional analyses should be explicitly marked exploratory.   The article addresses a relevant question and shows commendable transparency. However, methodological and interpretive revisions are required:   1. Reanalyse with count-based models (negative binomial with population offset). 2. Downplay or remove unstable regional/interaction models. 3. Clarify measurement limitations of law variables. 4. Reframe conclusions to avoid overstatement and highlight structural determinants. 5. Expand on diagnostics (collinearity, spatial autocorrelation, influence analysis). | We have removed Gamma GLM results in line with our new modelling strategy.  We have specified which countries were excluded.  We have advised caution in interpretation of the regional models.  Thank you very much – we believe we have addressed each of these overarching concerns by implementing the changes above. | Suppl. material |
| **Reviewer #2** |  |  |
| This study analyzes global legislation and regulation related to drowning prevention.  Please see my comments below.  Abstract  A scientific study should be replicable. In the abstract, the study type is unclear. Insufficient methodological details are given to the reader to understand what and how it was done? | Thank you for your helpful comments. We have responded to each specific piece of feedback below  Abstract – we have modified our abstract substantially, we hope this addresses your concerns. |  |
| Introduction  The introduction is written in an op-ed style. Most statements are without references. The literature search and knowledge gap are not clearly defined. | We have included further citations and better articulated the knowledge gap. As we noted above, there is a paucity of literature in this area. If things are still unclear we would be happy to consider more specific feedback on this. | Introduction |
| Novelty  This data is utilized from WHO report. What new information is it adding to the literature? | We put the WHO data into conversation with drowning rates alongside broader structural determinants of drowning a country level legislative analysis has not been undertaken before, nor was this done in the WHO reports. |  |
| Methods  Insufficient details regarding the 127 countries so we cannot understand who is included in this analysis. Grouping and interpreting high-Income countries and low-income countries together statistically is not the right approach to deduce meaningful results. | We have included a list of the countries included in the analysis.  While we group these countries in the main models, we control for things like GDP, so we are uncertain how to respond to this feedback. We would welcome more specific advice on what modelling approach may be appropriate here. As you will see we have provided substantive information on our methods, results and in supplementary material. | Methods |
| Results and Discussion  The findings identify known predictors of drowning, with negative results for legislative measures, ultimately not revealing anything meaningful and novel to add to the literature | We would encourage the reviewer to read our paper again. We believe that it is revealing that the legislative measures we included did not influence rates of drowning, and that several lessons can be taken away from this – we discuss some of these. | Results and discussion |
