# Supplementary material for: An analysis of global legislation and regulation related to drowning prevention
Source: PLOS Glob Public Health. 2026 Mar 25;6(3):e0005337. doi: 10.1371/journal.pgph.0005337 (PMC13016334; doi:10.1371/journal.pgph.0005337)
Supplement: Attachment — Submitted filename: Response_auresp_2.docx [file pgph.0005337.s019.docx]

| **Comment** | **Response** | **Location** |
| --- | --- | --- |
| **Reviewer 3** |  |  |
| Reviewer #3: This is an important study with global health implications. The authors used multiple sources of data and with a comprehensive set of structural covariates indicating major socioeconomic, environmental, and institutional determinants of drowning mortality. Also, for exposure, authors considered a variety of measures like the total count, enforcement strength, and their interaction an used multiple modeling strategies to model the relationship with exposure and outcome.  However, these are a few areas especially in design and methodology which if clarified or improved can further strengthen the paper. | Thank you very much for your kind feedback. We have responded to each point below. Should you have any further feedback we would be happy to consider this. |  |
| Aim 3:"whether strength of enforcement and institutional capacity were influential when it came to the effectiveness of legislation/regulation." Can authors possibly rephrase it in scientific and quantifiable language? | Thank you – we have changed this to read “3) whether strength of enforcement and institutional capacity were associated with the effectiveness of legislation/regulation” | Research aims |
| Measurement of Variables: Can authors elaborate how did they measure the extent to which legislative measures were enforced? A clear definition of the enforcement concept is crucial for interpreting the results.  Further, to enhance transparency, please provide a detailed list of the nine covariates included, categorized by their domains (e.g., economic, environmental).  Additionally, please provide an expanded explanation of the scoring mechanism used to derive the total number of legislative instruments (range 0-19) and the total enforcement score (range 0-190).  If possible, can authors include a table of Key explanatory variables, their data source, original form, transformation, and scoring.  Can authors spell time the abbreviation the first time they are used such as GDP PPP. Also, how legislative transparency and enforcement translates into V-DEM? | Thank you. We have now explained precisely what each of the enforcement measures is focused on and also provided citations in text if readers would like to explore further how each index was derived. As you will see, each is similar, but suitably different (we thought) to include as separate analyses.  In terms of the variables we included, we had already included a detailed table as supplementary material – with all variables, including the nine covariates included in modelling. Should there be anything more specific here we would be happy to consider it.  We have expanded our discussion re. scoring substantially.  Thank you – we believe this may already be addressed, both in our expanded discussion about data transformation and the existing table in supplementary material.  We have been more careful here and attempted to spell each abbreviation out on first use. We have also flagged readers to our table in supplementary material where full descriptions exist. | Methods |
| Reference and Citations: Please ensure full citations and/or direct links are provided for all external datasets, including the Varieties of Democracy (V-DEM), World Justice Project (WJP), Global Health Security (GHS), and the World Bank’s World Data Indicators (WDI).  Additionally, please provide specific references/citations to support the statement that the control variables were selected based on existing theoretical and empirical evidence on drowning prevention. | Thank you – we have now cited these.  We have now added a citation here. As you are probably aware there is a wealth of evidence that speaks to the environmental, economic and health-system related factors that are associated with drowning prevention. For this reason we have had to be selective with what we cite here. | Methods |
| Analytic Strategy: Please elaborate on the specific link function used for the outcome variable, which is the drowning mortality rate per 100,000 population. Although OLS was used, which assumes a linear outcome, given that the outcome is a rate derived from count data, please justify the OLS choice over alternative models like Poisson or Negative Binomial regression.  Furthermore, please detail the process for checking OLS assumptions (linearity, normality of residuals, etc.) and discuss the theoretical or empirical sources of heteroskedasticity that necessitated the use of robust variance estimators. | Our primary inference was based on negative binomial (NB2) regression models with a log link, which explicitly model drowning death counts with a population offset, properly accounting for the count nature of the data. We have now been more explicit about this in the paper.  While initial analyses (in our originally submitted paper) used OLS for interpretability, our primary inference in this revision is based on negative binomial (NB2) regression. We are therefore unsure of how to address this comment. We would be happy to consider further and more specific feedback however. | Methods |
| The stated goal 'to estimate the impact of legislation' implies a causal objective and thus the methods and language should be consistently aligned.  For example, if your goal was to estimate the impact of legislation on national drowning mortality, then legislation is your main primary exposure and has to be included in all nested models. I suggest authors read on Causal Inference in Epidemiology. See this for example: Daniel Westreich, Sander Greenland, The Table 2 Fallacy: Presenting and Interpreting Confounder and Modifier Coefficients, American Journal of Epidemiology, Volume 177, Issue 4, 15 February 2013, Pages 292–298, https://doi.org/10.1093/aje/kws412 and this: https://phlr.temple.edu/sites/phlr/files/documents/CPHLR-TheoryMethods2023_NaturalExperiments.pdf. | These are the stated aims of the paper and nowhere do we mention impact.  This study therefore sought to examine 1) the extent to which legislative measures, both individually and in aggregate, were associated with drowning mortality rates 2) the specific combinations of legal, environmental, health and governance factors that predicted countries with differential drowning burdens and 3) whether strength of enforcement and institutional capacity were associated with the effectiveness of legislation/regulation. Our analysis is ecological and cross-sectional.  Also, we make no claim re. causation in our discussion or in relation to our findings. While we do mention words like ‘effect’, we do not use this in relation to our findings. If there are instances of language which imply causation or are too strongly worded, we would be happy to revise this should you have further feedback.  We also appreciate your attention to causal inference principles. We are familiar with this work. We do not feel our modelling approach should raise concern here, but we do acknowledge our reporting of our findings could be improved. We have made changes here to focus more on the main effect and not interpreting covariates. We have also revised the manuscript to clarify that confounder coefficients are presented for completeness but not interpreted as causal effects. | Methods/ results |
| I see that authors have multiple aims and that authors used suite of different traditional regression and machine learning analysis. Can authors identify which analysis addressed which objective? Like Exploratory machine-learning classification of drowning risk addressed which objective? How aim 2 and 3 were addressed?  Also reconsider model specification again and if possible make a DAG to examine which variable are confounders (so called controls), mediator or collider? Because, if the included covariates (like GDP and urbanization) are colliders or mediators (depending on the specific causal pathway) introducing them could introduce bias or even mask exposure outcome relationship. | Thank you – we have now put this up front in our methods section.  We have added a DAG and subsequent sensitivity analyses to explore the influence of variables – these are reported over the methods, results and supplementary material. These results add further certainty to our variable selection | Methods/ results/ supplementary material |
| Results: Consider streamlining the presentation of results to focus on those that directly address the stated objectives. If authors think information in figure 1 and table 1 is overlapping, then chose to keep anyone of these in main paper and rest can go in supplementary information.  Second, for the interpretation of the main model, refer to paper by Westreich referred above, only interpretating the main effect.  Third, to provide essential context for the cross-national comparison, please include a table listing the included countries/geographies, alongside two key descriptive features: (1) a measure of inherent risk (e.g., proximity to water bodies, coastline length) and (2) their baseline legislative coverage (e.g., total count/score) | Thank you – we have not made substantive changes to our results (we felt it could become more confusing if we moved sections around), but we hope things are generally clearer now that we signpost which analyses answer each research question.  Please see our response above. We only focus on reporting the main effect in our amended results section.  Thank you. You will see we have updated/expanded table S3 to include some of this information. While we would love to be able to include things like waterbodies and coastline for example, we are unsure how this would be done within the scope of this paper – we assume this would involve some type of analysis involving satellite data. | Results / supplementary material |
| Interpretation/Discussion: Can authors address the possibility that the null finding of no effect of legislation on drowning could be an artifact due to methodology (design and analytic strategy) rather than conclusive evidence. | We have now acknowledged this more explicitly in our limitations section. | Limitations |
