# Supplementary material for: An analysis of global legislation and regulation related to drowning prevention
Source: PLOS Glob Public Health. 2026 Mar 25;6(3):e0005337. doi: 10.1371/journal.pgph.0005337 (PMC13016334; doi:10.1371/journal.pgph.0005337)
Supplement: Attachment — Submitted filename: Response_auresp_3.docx [file pgph.0005337.s020.docx]

| **Comment** | **Response** | **Location** |
| --- | --- | --- |
| **Reviewer 4** |  |  |
| -Clarify what kind of “null” this is (statistical vs. policy-relevant), why this matters, this paper is careful, but readers may still misread “no association” as “laws don’t matter." Authors can consider adding 2–3 sentences distinguishing statistical null findings at the ecological level from policy irrelevance (particularly emphasizing power, heterogeneity, and aggregation bias). | Thank you once again for your helpful and detailed feedback.  All of the changes we have made are highlighted in the manuscript.  We have added a further paragraph to our discussion section which further clarifies this. We are far more explicit now in making the point that our findings should not be interpreted as ‘laws don’t matter’ | Discussion |
| -To improve policy translation, the authors might briefly illustrate their findings with a concrete example (e.g., pool fencing in high-capacity vs. low-capacity settings, or alcohol regulation near water in differing enforcement contexts)..no new data required. Just narrative synthesis. | Thank you – we have now included some examples here, illustrating why having a law on paper often is not enough and that consideration should be given to things like resourcing and enforcement when it comes to legislation | Discussion |
| -Recommend authors adding a brief clarification that permutation importance reflects predictive contribution rather than causal influence, to prevent misinterpretation by non-technical readers. | This has now been clarified | Methods |
| -Consider highlighting earlier in the methods section that death counts were reconstructed from rates and population, and briefly noting how rounding error was handled (for method transparency and replication of the study). | We have no highlighted this earlier in the methods section and added further clarity on how this was rounded | Methods |
| -For data limitations, the authors could add few lines on how national-level aggregation may mask child-specific or occupational drowning effects where legislation is known to be effective.. | We have now done this | Discussion / limitations |
